# Non-Coding RNA in Salivary Extracellular Vesicles: A New Frontier in Sjögren’s Syndrome Diagnostics?

**DOI:** 10.3390/ijms241713409

**Published:** 2023-08-29

**Authors:** Tanya Cross, Kari Bente Foss Haug, Berit Sletbakk Brusletto, Stine Kamilla Ommundsen, Anne-Marie Siebke Trøseid, Trude Aspelin, Ole Kristoffer Olstad, Hans Christian Dalsbotten Aass, Hilde Kanli Galtung, Tor Paaske Utheim, Janicke Liaaen Jensen, Reidun Øvstebø

**Affiliations:** 1 The Regenerative Medicine Unit, Department of Medical Biochemistry, Oslo University Hospital, Ullevål, 0450 Oslo, Norway; 2 The Blood Cell Research Group, Department of Medical Biochemistry, Oslo University Hospital, Ullevål, 0450 Oslo, Norway; 3 Institute of Oral Biology, Faculty of Dentistry, University of Oslo, 0372 Oslo, Norway; 4 Department of Plastic and Reconstructive Surgery, Oslo University Hospital, 0372 Oslo, Norway; 5 Department of Ophthalmology, Oslo University Hospital, 0450 Oslo, Norway; 6 Department of Ophthalmology, Sørlandet Hospital Arendal, 4838 Arendal, Norway; 7 Department of Ophthalmology, Vestre Viken Hospital Trust, 3004 Drammen, Norway; 8 The Norwegian Dry Eye Clinic, 0369 Oslo, Norway; 9 Department of Oral Surgery and Oral Medicine, Faculty of Dentistry, University of Oslo, 0455 Oslo, Norway

**Keywords:** primary Sjögren’s syndrome, saliva, extracellular vesicles, RNA, non-coding RNA

## Abstract

Sjögren’s syndrome is an autoimmune rheumatic disease characterized by inflammation of the salivary and lacrimal glands, often manifesting as dry mouth and dry eyes. To simplify diagnostics of primary Sjögren’s syndrome (pSS), a non-invasive marker is needed. The aim of the study was to compare the RNA content of salivary extracellular vesicles (EVs) between patients with pSS and healthy controls using microarray technology. Stimulated whole saliva was collected from 11 pSS patients and 11 age-matched controls. EV-RNA was isolated from the saliva samples using a Qiagen exoRNeasy Midi Kit and analyzed using Affymetrix Clariom D™ microarrays. A one-way ANOVA test was used to compare the mean signal values of each transcript between the two groups. A total of 9307 transcripts, coding and non-coding RNA, were detected in all samples. Of these transcripts, 1475 showed statistically significant differential abundance between the pSS and the control groups, generating two distinct EV-RNA patterns. In particular, tRNAs were downregulated in pSS patients, with the transcript tRNA-Ile-AAT-2-1 showing a 2-fold difference, and a promise as a potential biomarker candidate. This study therein demonstrates the potential for using salivary EV-RNA in pSS diagnostics.

## 1. Introduction

Sjögren’s syndrome (SS) is a chronic autoimmune disease characterized by inflammatory destruction of the salivary and lacrimal exocrine glands with resultant hypofunction [1]. Consequently, patients present clinically with dry mouth (xerostomia) and dry eyes (xerophthalmia). Other extra-glandular manifestations may also occur. SS is referred to as primary SS (pSS) when it occurs alone [2] and secondary SS when diagnosed concurrently with another autoimmune condition, such as rheumatoid arthritis, systemic lupus erythematosus (SLE), or scleroderma [2]. The estimated worldwide prevalence of pSS is 0.06% [3]. pSS predominantly affects women, with a female-to-male ratio of 9:1 [2,4], and the incidence rate increases with age, peaking at 55–65 years for women [4].

While the exact etiology of pSS is still undetermined, a combination of environmental factors together with a genetic predisposition is presumed [2]. Viral infections have been considered a potential initiating cause, with a particular focus on the Epstein-Barr virus (EBV) [2,5].

The most recent set of classification criteria for pSS was developed based on an international collaborative effort by the American College of Rheumatology and the European League Against Rheumatism (ACR-EULAR) in 2016 [6]. These criteria require a score of ≥4 where a labial salivary gland biopsy with a focus score of ≥1 foci/4 mm^2^ gives 3 points. The presence of autoantibodies (anti-Ro/anti-SSA) also gives 3 points. An unstimulated whole saliva flow rate ≤ 0.1 mL/min, a Schirmer test ≤ 5 mm/5 min in at least one eye, and an ocular staining score ≥ 5 in at least one eye give 1 point each.

With no curative option presently available, treatment of pSS is generally a combination of targeted symptomatic therapy and broad-spectrum immunosuppressive therapy [7]. Early diagnosis is favorable for mitigating glandular inflammation and destruction. A specific test providing rapid diagnosis is thereby sought after. Saliva has gained interest as a potential source for pSS biomarkers [8]. As a biofluid, it has several advantages, including its relatively easy and non-invasive collection as well as being target-organ-relevant. There is additionally an emerging focus on using salivary extracellular vesicles (EVs) in pSS biomarker discovery, with EVs shielding their contents from enzymatic degradation [9] and thereby providing a more stable subset of molecules.

EVs are a heterogeneous group of membrane-bound, nano-sized particles released by cells into the extracellular space. They have been shown to participate in intercellular communication by means of either ligand-receptor binding or cellular uptake of EV bioactive components [9]. The main components of EVs are lipids, proteins, and nucleic acids, the composition of which reflects the cellular origin and mode of EV formation [9,10]. Analysis of the different constituents of EVs may therefore provide an insight into the pathophysiological state of cells, provide diagnostic modalities, and identify new therapeutic options.

Interest in EV-RNA analysis has increased with the knowledge that RNA transcripts can be transferred from cell to cell via EVs. Once delivered, the RNA transcripts are hypothesized to contribute to regulation within the recipient cell, thereby introducing the possibility of intercellular genetic communication. Advances in both next-generation sequencing (NGS) and microarray analysis, as well as improvements in bioinformatic data processing capabilities, have greatly facilitated the accumulation of EV-RNA data such that comparative studies are now possible. Comparing the EV-RNA profiles between pathological and healthy states may thereby uncover RNA transcripts as potential biomarker candidates. To date, there is minimal data available from salivary EV-RNA analysis in pSS patients [11,12,13].

The aim of this study was therefore to characterize the RNA content of salivary EVs using microarray technology and then compare the EV-RNA profiles between pSS patients and healthy controls.

## 2. Results

### 2.1. Extracellular Vesicle Characterization

**Figure 1:**
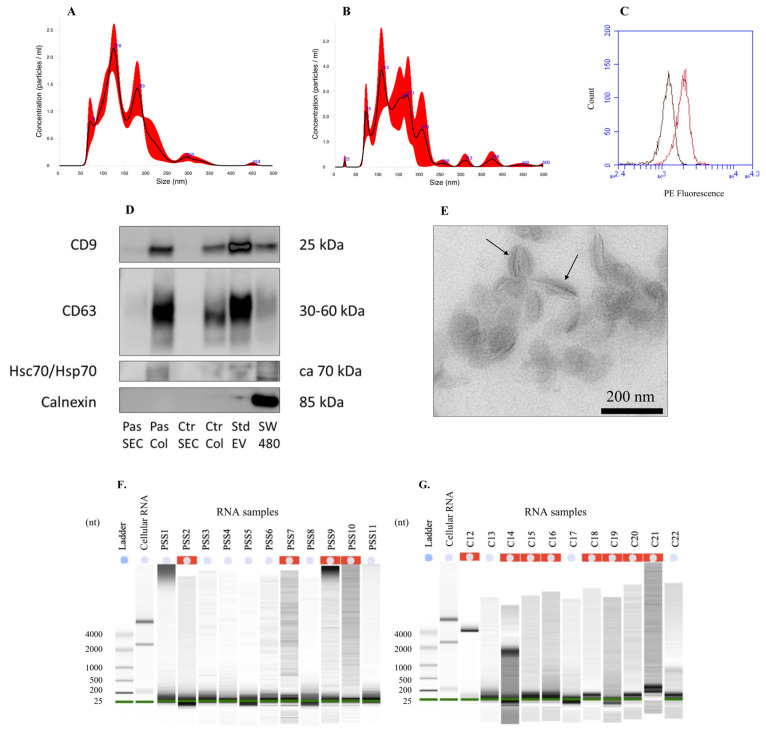
Characterization of salivary extracellular vesicles (EVs) and EV-RNA. (**A**) Size and concentration of EVs in the patient sample by nanoparticle tracking analysis (NTA). (**B**) Size and concentration of EVs in the control sample by NTA. (**C**) Flow cytometric detection of CD9-positive extracellular vesicles in the patient saliva sample. Red line: pSS patient; black line: isotype control. (**D**) Western blotting of EV-marker proteins. SEC = SEC EV isolation; Col = modified Qiagen exoRNeasy EV isolation; Pas = pSS patient; Ctr = healthy control; Std EV (recombinant EV standard) = positive control; SW 480 = positive control; CD9 and CD63 = membrane tetraspanins; Hsc70/Hsp70 = heat shock 70 protein, internal EV protein; Calnexin = endoplasmic reticulum protein; kDa = kilodalton. (**E**) A transmission electron microscopy (TEM) image of EVs in a pooled patient saliva sample. Arrows = cup-shaped EVs. Magnification 18,500×. Image courtesy of Espen Stang, Ph.D. (**F**) EV-RNA electropherograms, patient samples. (**G**) EV-RNA electropherograms, control samples. (nt) = nucleotides.

EVs were characterized in joint fractions 8–10 following SEC EV-isolation of a pooled pSS patient sample and a pooled control sample. NTA was used to determine the concentration and size distribution of EVs in the patient and control samples (Figure 1A,B). The pooled patient sample had a concentration of 2.0 × 10^9^ particles/mL, with a mean particle size of 154 nm, and the pooled control sample had a concentration of 3.7 × 10^8^ particles/mL, with a mean particle size of 157 nm.

The presence of the EV surface protein marker tetraspanin CD9 was assessed in the patient sample using flow cytometry (Figure 1C). The median fluorescence intensity (MFI) was 1946, with a shift in MFI of 764 relative to the isotype control, indicating an increased level of tetraspanin CD9 in the patient EV-isolated sample relative to the isotype control.

Western blotting to detect the EV tetraspanins CD9 and CD63, and the internal EV-marker protein heat shock 70 protein (Hsc70/Hsp70), was performed on EVs isolated from patient and control samples by both SEC and a modified Qiagen exoRNeasy column technique (Figure 1D). CD9 and CD63, as well as Hsc70/Hsp70, were detected in all four of the EV-isolated patient and control samples, although with reduced intensity in the SEC EV-isolated samples compared to the modified Qiagen exoRNeasy column EV-isolated samples. Furthermore, the negative protein control calnexin, an endoplasmic reticulum protein not found in EVs, was negative in all EV samples but positive in the SW480 cell lysate sample.

When viewed with TEM, EVs in the pooled pSS patient sample could be identified by their cup-shaped morphology and size (<220 nm). Some EVs were observed as aggregates (Figure 1E).

The average EV-RNA concentration of the patient samples was 3.5 ng/μL, with a range of 1.0 ng/μL to 10.0 ng/μL, according to Qubit^®^ 2.0 Fluorometer analysis. The average EV-RNA concentration of the control samples was 1.9 ng/μL, with a range of 1.0 ng/μL to 4.0 ng/L. Finally, the size distribution of the salivary EV-RNA was between 25 to 200 nucleotides in all samples, as demonstrated by Agilent 2100 Bioanalyzer analysis (Figure 1F,G).

### 2.2. Salivary EV-RNA Analysis Using Affymetrix Clariom™ D Microarrays

Both coding RNA (mRNA) and non-coding RNA (ncRNA) were detected in all saliva samples. A total of 9307 transcripts were identified from the microarrays’ total of approximately 540,000 transcripts (1.7% transcripts identified). Transcript signal values ranged from under 10 to over 11,000.

**Figure 2:**
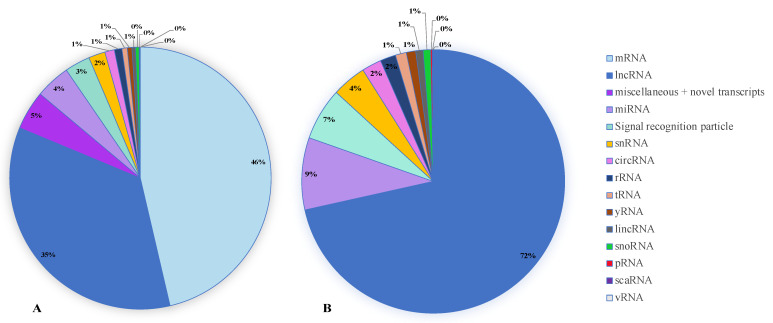
Categorization of the RNA transcripts identified in salivary EVs by Affymetrix Clariom™ D microarray analysis. (**A**) All RNA transcripts detected (9307). (**B**) Non-coding RNA (ncRNA) subtypes only (no mRNA and no miscellaneous or novel transcripts) (4543). A value of 0% indicates a value between 0 and 1%. mRNA = messenger RNA; lncRNA = long non-coding RNA; miRNA = microRNA; snRNA = small nuclear RNA; circRNA = circular RNA; rRNA = ribosomal RNA; tRNA = transfer RNA; yRNA = Y RNA; lincRNA = long intergenic RNA; snoRNA = small nucleolar RNA; pRNA = promoter-associated RNA; scaRNA = small Cajal body-specific; vRNA = vault RNA.

Most known RNA subtypes were identified in all the patient and control saliva samples (Figure 2A). Close to half of the RNAs detected were mRNA (46%), and close to half were ncRNAs (46%). Miscellaneous and novel transcripts (5%) and signal recognition particles (3%) comprised the remaining 8%. Long non-coding RNA (lncRNA) represented the most abundant ncRNA subtype, comprising 72% of the total (Figure 2B).

#### 2.2.1. Comparing Affymetrix Microarray Results between pSS Patient and Control Groups

**Figure 3:**
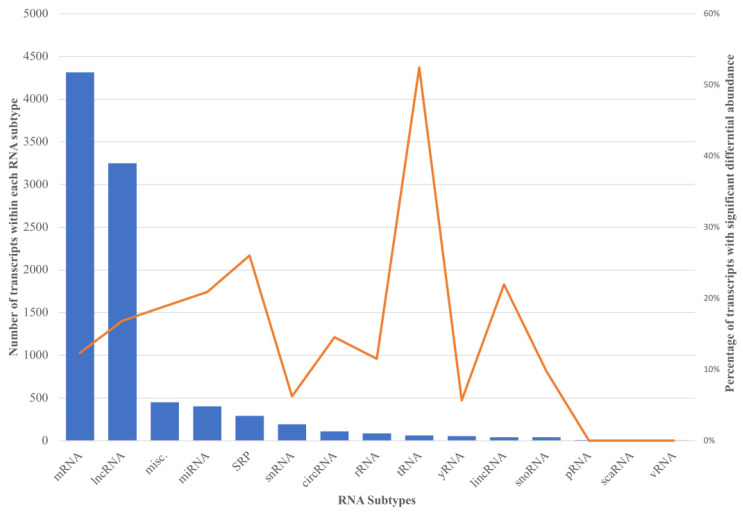
Dual comparison of RNA subtypes. The number of transcripts detected within each RNA subtype (blue columns) and the corresponding percentage of transcripts within each subtype with statistically significant differential abundance between the pSS and control groups (orange line), *p*-value < 0.05. SRP = signal recognition particle RNA; misc. = miscellaneous transcripts.

A one-way ANOVA was used to compare the mean fluorescence signal values, representing the mean transcript levels, of the pSS patient and control transcripts. Of the 9307 transcripts detected, 1475 had a statistically significant differential abundance between the two groups, *p*-value < 0.05, and approximately 60% of these were ncRNA. After controlling statistically for the false discovery rate (FDR), seven of these transcripts showed *q*-values < 0.05, where five were lncRNAs, one a tRNA (tRNA-Ile-AAT-2-1), and one a miRNA (MIR6870). The percentage of transcripts showing statistically significant differential abundance within each RNA subtype is displayed in Figure 3, with tRNA having the most pronounced outcome, with over 50% of the transcripts showing a significant difference.

**Figure 4:**
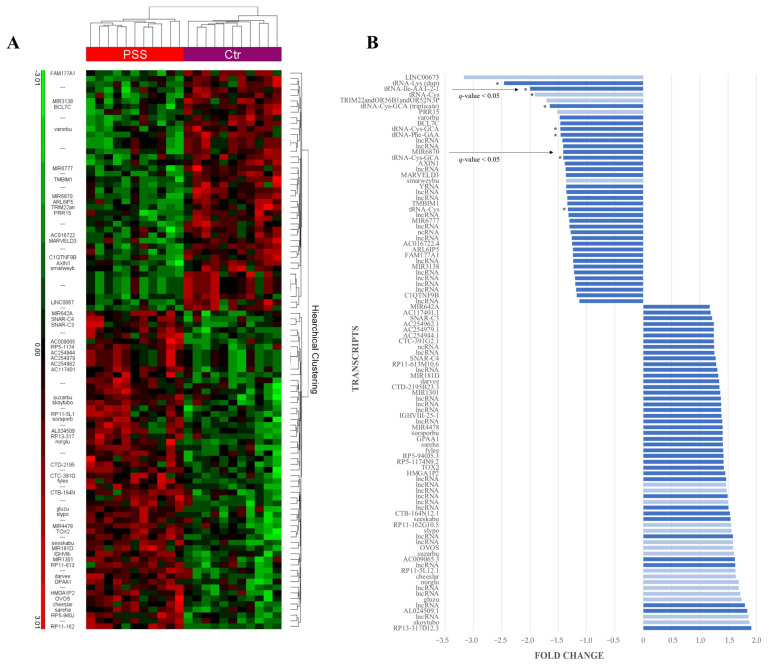
The 100 most statistically significant differentially abundant transcripts between the patient (pSS) and control groups (Ctr), *p*-value < 0.05. (**A**) A heatmap displaying hierarchical clustering of the transcripts. Green represents a lower level in one group relative to the other, and red vice versa. PSS: pSS patient group; Ctr: control group; blank or ---: unannotated transcripts. (**B**) Transcripts from the heatmap are displayed and ordered according to their fold change. Dark blue columns: transcripts with mean signal values ≥20; light blue columns: transcripts with mean signal values < 20. * tRNA transcripts; arrows: transcripts with *q*-value < 0.05.

A hierarchical cluster heatmap was produced, using the 100 transcripts with the most statistically significant differences between the patient and the control groups (Figure 4A). Of these transcripts, 47 are not yet functionally annotated but are inferred to be lncRNAs based on their database source. The 100 transcripts were further explored and plotted according to their fold change (Figure 4B). Transcripts with signal values above and below 20 were distinguished, with a value ≥ 20 chosen as a measure of biological significance. The list was adjusted to 97 after identifying a triplicate and a duplicate set of transcripts. Of these, 30 transcripts showed a fold change ≥ 1.5. Eight tRNA transcripts identified had reduced levels in the patient group relative to the control group. The three transcripts having both a fold change ≤ −1.5 and a signal value ≥ 20 were all tRNAs: tRNA-Lys, tRNA-Ile-AAT-2-1, and tRNA-Cys-GCA, with tRNA-Ile-AAT-2-1 also having a *q*-value < 0.05. LINC00673 was identified as the transcript with the greatest negative fold change (−3.2), however, the mean signal values were <20. Of the six miRNAs included, MIR6870 had a *q*-value < 0.05, with a fold change of −1.4.

**Figure 5:**
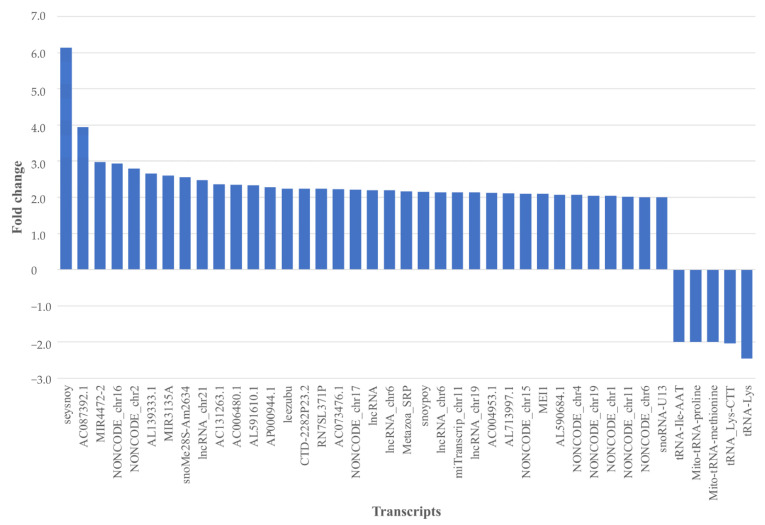
Transcripts with statistically significant differential abundance between the pSS patient and control groups with a fold change of 2 or more, and a signal value above 20, *p*-value < 0.05. Positive fold change = higher transcript level in the pSS patient group relative to the control group, and vice versa. Mito-tRNA: mitochondrial tRNA. Transcripts depicted by NONCODE are miscellaneous or novel transcripts identified as non-coding RNA (ncRNA) but with unknown functions.

In order to further distinguish transcripts with a difference in abundance between the patient and the control groups, transcripts with statistically significant differential abundance were further filtered for fold change (≥2) and mean signal values (≥20) (Figure 5). The mRNA transcript ‘seysnoy’ was identified as having a fold change greater than six, with a higher level seen in the patient group relative to the control group. Moreover, the transcript with gene symbol AC087392.1 had a positive fold change close to 4. Furthermore, the immature miRNAs MIR4472-2 and MIR3135A both showed a positive fold change > 2. All five transcripts showing a negative fold change were tRNAs, two of mitochondrial origin: tRNA-Ile-AAT-2-1, mitochondrially encoded tRNA proline, mitochondrially encoded tRNA methionine, tRNA-Lys-CTT-4-1, and tRNA-Lys.

#### 2.2.2. RNA Validation

RT-qPCR was used to validate two of the RNA transcripts, B2M and RPL9, in three pSS patients and three control samples. The RT-qPCR results confirmed transcript levels consistent with the different signal values detected by the microarrays.

## 3. Discussion

The Affymetrix microarray analysis of salivary EV-RNA unveiled an extensive repertoire of coding and non-coding RNAs in all samples. Approximately half of the RNA transcripts identified were mRNAs, while the other half were ncRNAs, of which most known ncRNA subtypes were detected. These included lncRNA, miRNA, snRNA, circRNA, rRNA, tRNA, yRNA, lincRNA, snoRNA, pRNA, scaRNA, and vRNA. A comparison of the RNA profiles between patients with pSS and controls revealed statistically significant differential abundance in the levels of approximately 16% of transcripts. The most remarkable difference was seen in tRNA transcripts, with close to 50% showing a significant difference between the patient and control groups. Several mRNA and miRNA transcripts also deserve further consideration. Furthermore, an abundance of lncRNAs was detected, five with a *q*-value < 0.05.

To our knowledge, Ogawa et al., (2008) were the first to show that human whole saliva contains EVs [14]. Subsequently, 509 mRNA transcripts were identified in salivary EVs isolated from healthy individuals, using microarray analysis [15]. This study also observed the transfer of mRNA from EVs to target cells when incubating oral keratinocytes with salivary EVs and further demonstrated that EVs provide RNA with protection against salivary nucleases. These observations were in line with other studies showing that both mRNA and miRNA are transferable in EVs and can have functional capabilities in recipient cells [16,17,18,19], thereby providing the basis for the current study.

### 3.1. A Comparison of the mRNA Transcripts in Salivary EVs between pSS Patients and Controls

mRNAs represented approximately half of the RNA transcripts detected by the microarrays, with almost 12% showing statistically significant differential abundance between the pSS and control groups. Of these, two stood out as transcripts of interest: seysnoy and AC087392.1. These transcripts had positive fold changes of 6 times and close to 4 times, respectively, with higher levels of the transcripts in the pSS patient group compared with the control group.

The official symbol for the seysnoy gene is MTRNR2L2 and it is located on chromosome 5. While there is uncertainty as to whether it is a transcribed protein-coding gene or a paralog of the mitochondrial gene MT-RNR2, several recent studies have identified its potential involvement in coronary artery disease, tumorigenesis, Huntington’s disease, and diabetic kidney disease [20,21,22,23,24].

There are presently no disease or trait phenotypes found in connection with the protein-coding gene AC087392.1. Further investigation of this transcript is warranted.

### 3.2. A Comparison of the miRNA Transcripts in Salivary EVs between pSS Patients and Controls

In this study, immature miRNAs represented 9% of the ncRNA transcripts detected in salivary EVs, with 21% of the identified miRNAs showing a significant difference in abundance between the two groups. MiRNAs have a well-established role in translational and transcriptional regulation, and their dysfunction has been linked to many pathological conditions, including neurodegenerative disorders, cardiovascular disease and obesity [25], autoimmune diseases [26], and various forms of cancer [27,28]. In the present study, both MIR4472-2 and MIR3135A were shown to have statistically significant differential abundance between pSS patients and controls with enriched levels in the patient group and fold change differences of 3 and 2.6, respectively. However, the biological significance of these findings must be further evaluated at the miRNA level since the chosen microarrays did not use functionally recognized mature miRNA.

### 3.3. A Comparison of the tRNA Transcripts in Salivary EVs between pSS Patients and Controls

Close to 50% of the 55 different tRNA transcripts detected in the salivary EV-RNA samples showed statistically significant differential abundance between the pSS patient and control groups. Five showed a fold change over two and when correcting for the false discovery rate, and tRNA-Ile-AAT-2-1 had a *q*-value < 0.05. Follow-up validation is required using either RT-qPCR or NGS.

There are over 600 tRNA loci [29,30] positioned at multiple sites throughout the genome, coding for a possible 64 anticodons. The largest collection of tRNA genes, with 157 genes, is found on chromosome 6 [30]. There are additionally mitochondrial tRNA genes and tRNA pseudogenes [30]. While full-length tRNAs are recognized for their role in binding amino acids and translating mRNA in protein production, fragments derived from tRNAs are believed to contribute a whole new division to the realm of the regulatory ncRNAs [31]. Lee et al. were one of the first to suggest that tRNA fragments (tRFs) are a new group of short ncRNAs that are not merely the result of tRNA degradation or a by-product of biogenesis [29]. The results from this current study do not, however, distinguish between whole tRNAs and tRFs, as transcript fragmentation is central to the preparation of RNA for microarray analysis. Sequencing of salivary EV tRNA would perhaps provide a more complete picture. That said, it is important to note that tRNAs are heavily base modified, and this in turn affects the sequencing [32,33].

tRNA fragments are proposed to have roles in various cellular processes, including cell proliferation [32,33], RNA interference [32,34], and protein synthesis [32]. They have accordingly been linked to tumorigenesis, neurodegenerative disorders, metabolic diseases, the response to stress, and viral infections [33,35,36,37,38,39,40,41]. Interestingly Li et al. were the first to publish results from a clinical trial that discovered the tRF tRNA-Gly-GCC-5 in salivary EVs as a potential biomarker for esophageal carcinoma [42]. With mounting evidence for the biomarker potential of EV tRFs, the results from this study indicate that further research into salivary EV tRNAs is merited.

### 3.4. A Comparison of the yRNA Transcripts in Salivary EVs between pSS Patients and Controls

A total of 53 yRNA transcripts were identified in the salivary EV-RNA samples. Four of the transcripts were annotated as Ro-associated Y1, Y3, Y4, and Y5, originating from chromosome 7. Interestingly, none of these four transcripts showed a statistically significant difference in abundance between the patients and controls. The remaining yRNA transcripts were categorized as pseudogenes, originating from diverse chromosomes, with three showing a statistically significant difference in abundance between the patient and control groups, but with fold changes < 1.5.

Y-RNAs were originally discovered as the RNA component of ribonucleoprotein (RNP) complexes in the serum of patients with pSS and SLE. They were found bound to the RNA-binding proteins Ro60 and La, which are now considered the main autoantigenic targets in both pSS and SLE [43]. Y-RNAs are evolutionary conserved small RNAs, 83–112 nt in length [44]. Humans have four different types, Y1, Y3, Y4, and Y5, which are all encoded by single-copy genes clustered on chromosome 7 [45]. The additional yRNA pseudogenes are located at various positions throughout the genome [45]. The binding of Y-RNA to Ro60 and La facilitates either nuclear retention or nuclear export, as well as enhancing yRNA stability [43]. So far, it is known that they are involved in DNA replication, ncRNA quality control, and response to cellular stress [44,45,46]. Evidence also supports the selective inclusion of Y-RNAs into EVs [47,48]. Furthermore, the Y-RNA-Ro/La RNP complexes have also been identified in EVs originating from salivary gland epithelial cells [49], with the authors of the article suggesting that this could be a method by which “intracellular autoantigens are presented to the immune system with an immunogenic or tolerogenic outcome”.

Considering the occurrence of autoantigens to Ro60 and La in the majority of pSS patients, and the association these antigens have with Y-RNA, one could conceivably expect to see a discrepancy in the EV-associated Y-RNAs between pSS patients and controls. Interestingly, the present study found that the levels of all four Y-RNA types were present at near equal levels in the two groups, with the highest levels found for Y3, followed by Y1, Y4, and then Y5.

While it is beyond the scope of this article to make any predictions regarding the involvement of Y-RNA-RNP complexes and autoimmunity in SS, it is noteworthy that Y-RNA, as well as signal recognition peptide- (SRP) RNA, are also selectively incorporated into the capsids of certain viruses [48]. Moreover, it has been postulated that an immune response to Ro60-positive commensal bacteria in pSS patients could be an alternate manner in which autoimmunity is established [50].

### 3.5. A Comparison of the lncRNA Transcripts in Salivary EVs between pSS Patients and Controls

Of the ncRNA transcripts identified by the microarray analysis, over 70% were lncRNA. Of these, 17% showed statistically significant differential abundance between the patient group and the control group. However, most of the lncRNA transcripts detected by the microarrays were transcripts with an uncharacterized function and with no gene symbol. At least 42 of the transcripts included in the heatmap were lncRNAs, five with a *q*-value < 0.05. While the function of lncRNAs has not been definitively determined, they are believed to be involved in the regulation of gene expression [51].

### 3.6. RNA in EVs—General Considerations

Comparisons between EV studies are difficult due to the lack of standardization of methods. Both the EV isolation method and the EV-RNA isolation method, as well as the type of RNA analysis, greatly impact the RNA transcripts identified. In the future, improved and standardized EV-RNA isolation techniques may provide more comparable RNA samples. Increasing the depth of EV-RNA sequencing will similarly broaden the probe options available for microarray production.

While NGS techniques enable the analysis of the entire genetic material of a sample, microarrays provide a method for profiling previously identified nucleic acid sequences. This presents the obvious disadvantage that novel sequences will not be identified [52]. However, a significant advantage of microarrays is that the results do not require as elaborate post-analytical bioinformatics as NGS [52]. Furthermore, whole saliva also has a large microbiota. Microarray analysis of salivary EV-RNA simultaneously captures a defined sub-population of extracellular RNA and specifically detects human-only RNA [53].

The Clariom™ D microarrays used in this study were designed for use with total cellular RNA. Only 1.7% of the approximate 540,000 transcripts represented on the chips hybridized with the RNA transcripts from the salivary EVs. There are several apparent explanations for this, but they are yet unsubstantiated. Firstly, it supports the notion that EV-RNA is a selection of total cellular RNA. Secondly, the concentration of EV-RNA is substantially less than cellular RNA. Thirdly, the Affymetrix microarrays are designed for, and the hybridization steps are optimized for, total cellular RNA, not EV-RNA. Taking this into consideration, the EV transcripts identified should only be considered a representation of the total.

Most of the RNAs in EVs are under 200 nt in length [54,55]. The results from the Bioanalyzer analysis performed in this study were in accordance with this. While many regulatory ncRNAs are shorter than 200 nt, both mRNA and lncRNA generally have original transcript lengths over 200 nt. It can therefore be surmised that the majority of these latter transcripts were in fragmented form in the salivary EVs. What is not known, however, is if the fragmentation is due to deliberate cleavage or degradation.

## 4. Materials and Methods

**Figure 6:**
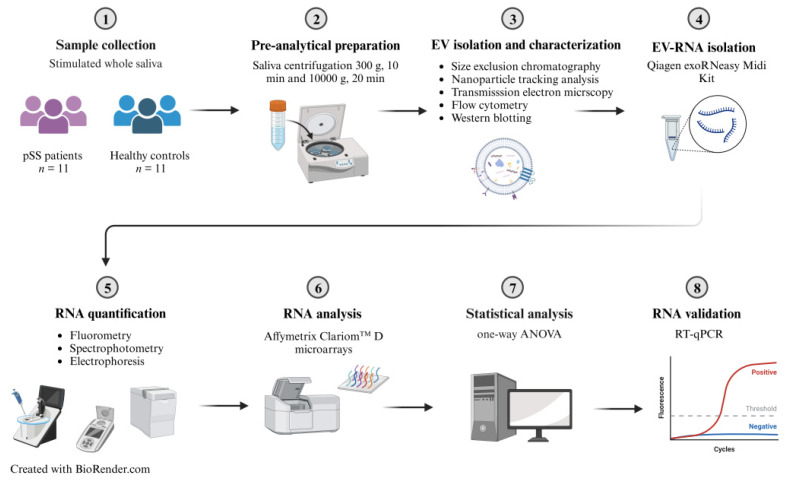
Study workflow.

An overview of the study workflow is illustrated in Figure 6.

### 4.1. Study Participants and Saliva Collection

Saliva samples were provided by the Dry Mouth Clinic at the Department of Clinical Dentistry, Faculty of Dentistry, University of Oslo. Stimulated whole saliva (SWS) was collected from 11 pSS patients and 11 age-matched healthy controls. All participants were women aged between 39 to 72 years, with a mean age of 55. All pSS patients fulfilled the ACR criteria; all were ANA- and anti-SSA-positive and had dry eyes, dry mouth, and reduced saliva secretion (Table S1). Most of the patients were also anti-SSB-positive (8) and had reduced tear secretion (9), while 1 did not tolerate the Schirmer test. Most of the patients were not subjected to salivary gland biopsies, as they already fulfilled the various criteria sets. The control individuals were healthy subjects experiencing no symptoms of dry mouth or dry eyes, having no SS-associated disease condition, and using no medication with influence on saliva secretion. No eating or drinking was permitted for the hour prior to saliva collection. SWS was collected for five minutes by the subjects spitting into a collection cup placed on ice while chewing on a paraffin pellet (Ivoclar Vivadent, Shaen, Lichtenstein). Aliquots with volumes of approximately 500 μL, were stored at −80 °C.

### 4.2. Preanalytical Sample Preparation

Saliva samples were thawed on ice and then, to inhibit enzymatic activity, 1 μL of RiboLock RNase Inhibitor (40 U/μL) (Thermo Fisher Scientific, Oslo, Norway), together with 5 μL of cOmpleteTM Mini Protease Inhibitor (Roche), were added per 1 mL of saliva and mixed well. Filtered phosphate-buffered saline (PBS) (0.1 μm filter, Millex^®^-GV, Merck Millipore, Cork, Ireland) was subsequently added to each sample at a ratio of 1:1. Two centrifugation steps were performed: 300 g for 10 min, 4 °C, to remove cells, bacteria, and food residue, and 10,000× *g* for 20 min, 4 °C, to remove cellular debris and other particulate matter. Next, 500 μL of each sample was mixed with an equal volume of filtered PBS and then filtered with a 0.22 μm filter (Millex^®^-GV, Merck Millipore, Cork, Ireland).

### 4.3. EV Characterization

Due to limited patient material, EV characterization was performed on pooled samples. EVs were isolated from pooled patient saliva samples (*n* = 11) and pooled control saliva samples (*n* = 11) using size-exclusion chromatography (SEC) with IZON qEV original 70 nm (Izon Science, Lyon, France) isolation columns. Pooled samples (500 μL) were diluted 1:1 with filtered PBS (0.1 μm), applied to pre-washed SEC columns, and EVs eluted into 10 fractions of 500 μL. Combined joint fractions 8–10 were further used for EV characterization.

Due to the relatively low EV yield following SEC-EV isolation, EVs were also isolated using a modified Qiagen exoRNeasy EV isolation technique for use in Western blotting. Briefly, 200 μL of pooled patient saliva (*n* = 4) and 200 μL of pooled control saliva (*n* = 4) were used in the first spin column steps of the exoRNeasy protocol to isolate and capture the EVs in the column filters; see Section 4.4. EVs were then lysed with 100 μL of a RIPA buffer/protease-inhibitor mix (RIPA 5× Buffer, Thermo Fisher Scientific, Oslo, Norway/cOmplete, Mini, EDTA-free Protease Inhibitor Cocktail 25×, Roche, Oslo, Norway). The eluates were then used for Western blotting together with filter-concentrated SEC-isolated EV samples. SEC-isolated EV samples were concentrated from 250 μL to 100 μL using Amicon^®^ Ultra-2, 100 K (Merck Millipore, Cork, Ireland).

The size distribution and particle concentration of EVs in the pSS and control samples were examined by nanoparticle tracking analysis (NTA) using a Nanosight NS500 Instrument (Malvern Instruments Ltd., Amesbury, UK) [56], with detection threshold 3 and camera level 14.

Transmission electron microscopy (TEM) was performed on the patient samples. Formvar-carbon coated copper grids were placed on top of 5 μL drops of sample, incubated for 5 min at RT, washed 3 times with distilled H_2_O, incubated with 2% methylcellulose containing 0.3% uranyl acetate for 10 min on ice, and then air-dried. A Tecnai G2 Spirit transmission electron microscope (FEI, Hillsboro, OR, USA) was used together with a Morada digital camera and RADIUS imaging software (version 2.1) to create images which were further processed using Adobe Photoshop.

Immunoaffinity capture and detection of EV-membrane protein CD9 were performed in conjunction with flow cytometry to identify EVs in the patient sample, as previously described [56]. EVs were captured with the Exosome-Human CD9 Flow Detection Reagent (cat. no. 10620D, Thermo Fisher Scientific, Oslo, Norway) and the captured EVs were stained with anti-CD9-phycoerythrin (PE) (cat. no. 555372, BD Biosciences, Oslo, Norway) or isotype-matched control (IgG1-PE, cat. no. 559320, BD Biosciences, Oslo, Norway). The median fluorescence intensity (MFI) of each sample was recorded and the ΔMFI was calculated by subtracting the MFI of the isotype control from the sample MFIs.

Western blotting was performed as previously described [56]. In addition to the four salivary EV-isolated samples (SEC- and exoRNeasy isolated EVs), a recombinant Exosome standard (Merck Life Science, Oslo, Norway, Cat no SAE0193) and an SW480 cell lysate were used as controls. Primary antibodies were used against recognized EV markers: anti-CD9 1:750 (Invitrogen, Thermo Fisher Scientific, Oslo, Norway, Cat no 10626D), anti-CD63 1:600 (Invitrogen, Cat no 10628D), anti-Hsc70/Hsp70 1:1000 (Enzo Life Science, AH Diagnostics AS, Oslo, Norway, Cat no ADI-SPA-820), and anti-Calnexin 1:1000 (Abcam, Cambridge, UK, Cat no ab22595). SeeBlue^®^ Plus 2 Pre-Stained Protein Standard (Thermo Fisher Scientific, Oslo, Norway) was used as a ladder.

### 4.4. EV-RNA Isolation Using a Qiagen exoRNeasy Midi Kit

EV-RNA isolation was performed on all individual saliva samples using a Qiagen exoRNeasy Midi Kit (Qiagen, Hilden, Germany), according to the manufacturer’s guidelines. Sample volumes of 500 μL were applied to the Qiagen exoRNeasy isolation columns and 200 μL of the aqueous phase was transferred. Additionally, a DNase step was included following the 100% ethanol-centrifugation step. RNA was recovered using RNAse-free water, producing elutes of approximately 12 μL, and subsequently quantified and characterized. The samples were frozen at −80 °C until further use.

### 4.5. RNA Quantification, Purity Assessment, and Characterization

A Qubit^®^ 2.0 Fluorometer (Life Technologies Corporation, Carlsbad, CA, USA) was used in conjunction with a Qubit^®^ microRNA Assay Kit (Invitrogen by Thermo Fisher Scientific, OR, USA) to determine the RNA concentration of all salivary EV-RNA samples. A Nanodrop™ One Spectrophotometer (Thermo Fisher Scientific, Oslo, Norway) was used to assess the presence of potential contaminants (guanidine or phenol) and a Bioanalyzer (Agilent Technologies, Santa Clara, CA, USA) analysis was performed on all samples using an Agilent 6000 Pico Assay Kit (Agilent Technologies, Santa Clara, CA, USA) to assess the size and quality of the RNA. All analyses were performed according to the manufacturer’s guidelines and using 1 μL of sample.

### 4.6. RNA Analysis Using Affymetrix Clariom™ D Microarrays

Clariom™ D microarray chips (Thermo Fisher Scientific, Oslo, Norway) were used to analyze the salivary EV-RNA samples. The samples were prepared using a GeneChip^TM^ WT Pico Reagent Kit (Thermo Fisher Scentific, Oslo, Norway, Cat no 902623), in accordance with the manufacturer’s guidelines. The input quantity of total RNA was 3 ng and pre-IVT amplification was performed using 9 PCR cycles. Hela RNA (0.5 ng total RNA) was used as a positive control to verify that the reagents worked as expected, and RNA-free water was used as a negative control to monitor for RNA and DNA contamination. Loaded Clariom^TM^ D microarrays were incubated in an Affymetrix GeneChip™ 645 hybridization oven for 17 h at 45 °C with a rotation of 60 rpm, then washed and stained in a GeneChip™ Fluidics Station 450 using a GeneChip™ Hybridization, Wash, and Stain Kit (Thermo Fisher Scientific, Oslo, Norway, Cat no 901241). A GeneChip^™^ Scanner 3000 System was used to read the generated fluorescence signal values, corresponding to RNA transcript levels, and scanned data were processed with Affymetrix Command Console^®^ Software version 4.0 (AGCC), producing DAT files ready for bioinformatic processing (all instruments provided by Affymetrix, Santa Clara, CA, USA).

### 4.7. RNA Validation Using RT-qPCR

Reverse transcription-quantitative PCR (RT-qPCR) was used to validate the presence of selected RNA transcripts (Ribosomal Protein L9, RPL9, and beta-2-microglobulin, B2M) based on the availability of assays in the laboratory and sufficiently high and even signal values across all the samples applied to the arrays (RPL9 Taqman^®^ Gene Expression Assay, Hs01552541_g1 and B2M Taqman^®^ Gene Expression Assay, Hs99999907_m1). A Taqman™ Fast Advanced Master Mix (ref 4444557) was used. Three patient samples and three control samples were used to generate the cDNA library with SuperScript™ Enzyme Mix (Cat no 11754-050) and VILO™ Reaction Mix (Cat no 11754-250). Total cellular RNA and RNA-free water were used as positive and negative controls, respectively. All reagents were provided by Thermo Fisher Scientific, Oslo, Norway.

### 4.8. Bioinformatic Processing

Partek^®^ Genomics Suite^®^ software (version 6.6) was used for the normalization and statistical analysis of the Affymetrix microarray results. The DAT files were normalized using Robust Multichip Average (RMA) normalization and the resulting CEL files were filtered to remove transcripts with signal values under 5, thereby reducing background interference. A one-way Analysis of Variance (ANOVA) was then performed to determine any statistically significant difference in transcript levels between the patient and control groups, with *p*-values less than 0.05 considered indicative of statistical significance. *q*-values and fold changes were also determined. Additionally, a Principal Component Analysis (PCA) and a hierarchical cluster heatmap were produced, with the heatmap incorporating the 100 transcripts with the most statistically significant differences in signal value between the groups.

## 5. Conclusions

This present study uncovered several differentially abundant salivary EV-RNA transcripts between pSS patients and controls. These transcripts included both mRNAs and ncRNAs, specifically miRNAs, tRNAs, and lncRNAs. The most striking finding was the differential abundance of tRNA transcripts between the groups, in particular, transcript tRNA-Ile-AAT-2-1, indicating their potential use in biomarker discovery.

## Data Availability

Data can be obtained from the corresponding author upon request.

## Supplementary Materials

The following supporting information can be downloaded at: https://www.mdpi.com/article/10.3390/ijms241713409/s1.
