# Supplementary material for: Non-Coding RNA in Salivary Extracellular Vesicles: A New Frontier in Sjögren’s Syndrome Diagnostics?
Source: Int J Mol Sci. 2023 Aug 29;24(17):13409. doi: 10.3390/ijms241713409 (PMC10488010; doi:10.3390/ijms241713409)
Supplement: Supplementary file 1 [file ijms-24-13409-s001.zip › ijms-2561434-supplementary.pdf]

International Journal of
Molecular Sciences

MDPI

Article

Non-Coding RNA in Salivary Extracellular Vesicles: A New Frontier in Sjögren's Syndrome Diagnostics?

Supplementary Materials

Table S1. Clinical data of pSS study participants.

ID Number	Age [1] (Years)	Anti-SSA [2]	Anti-SSB [2]	Dry Mouth [3]	Dry Eyes [4]	Saliva Secretion [5]	Schirmer Test [6]
PSS1	55	+	+	+	+	+	+
PSS2	64	+	+	+	+	+	+
PSS3	72	+	+	+	+	+	*
PSS4	47	+	+	+	+	+	+
PSS5	68	+		+	+	+	+
PSS6	48	+	+	+	+	+	+
PSS7	44	+	+	+	+	+	+
PSS8	57	+	+	+	+	+	
PSS9	71	+		+	+	+	+
PSS10	48	+	+	+	+	+	+
PSS11	39	+		+	+	+	+

[1] Age = age at biopsy

[2] Serum autoantibodies, + indicates positivity

[3] Patient-reported symptoms of dry mouth, + indicates dryness

[4] Patient-reported symptoms of dry eyes, + indicates dryness.

[5] Unstimulated salivary secretion, normal salivary flow > 1.5ml/15min, + indicates ≤ 1.5ml/15min

[6] Tear secretion, normal tear flow > 5mm/5min, + indicates ≤ 5mm/5min

* The patient did not tolerate the Schirmer test

pSS: Primary Sjögren's Syndrome

Anti-SSA: anti- Sjögren's Syndrome-related antigen A autoantibodies

Anti-SSB: anti- Sjögren's Syndrome-related antigen B autoantibodies.
